# Yield, Chemical Composition and Bioactivity of Essential Oils from Common Juniper (*Juniperus communis* L.) from Different Spanish Origins

**DOI:** 10.3390/molecules28114448

**Published:** 2023-05-30

**Authors:** Luis Saúl Esteban, Irene Mediavilla, Virginie Xavier, Joana S. Amaral, Tânia C. S. P. Pires, Ricardo C. Calhelha, César López, Lillian Barros

**Affiliations:** 1 Centre for the Development of Renewable Energies—Centre for Energy, Environmental and Technological Research (CEDER-CIEMAT), Autovía de Navarra A-15, Salida 56, 42290 Lubia, Spain; irene.mediavilla@ciemat.es; 2 Centro de Investigação de Montanha (CIMO), Instituto Politécnico de Bragança, Campus de Santa Apolónia, 5300-253 Bragança, Portugal; virginie.xavier@ipb.pt (V.X.); jamaral@ipb.pt (J.S.A.); tania.pires@ipb.pt (T.C.S.P.P.); calhelha@ipb.pt (R.C.C.); lillian@ipb.pt (L.B.); 3 Laboratório Associado para a Sustentabilidade e Tecnologia em Regiões de Montanha (SusTEC), Instituto Politécnico de Bragança, Campus de Santa Apolónia, 5300-253 Bragança, Portugal; 4 Department of Systems and Natural Resources, ETSI Montes, Forestal y del Medio Natural, Edif. Forestales, Polytechnical University of Madrid, C/José Antonio Novais, 10, 28040 Madrid, Spain; cesar.lopez@upm.es

**Keywords:** distillation, foliage, essential oil, antioxidant activity, cytotoxicity, anti-inflammatory activity

## Abstract

Essential oils (EOs) obtained from *Juniperus communis* L. are frequently used in the production of bioproducts. However, there are no studies regarding industrial crops’ production, allowing for better control of the quality and production of juniper EOs. To select the plant material for developing future crops of this species in northern Spain, four locations where this shrub species grows in the wild were selected and samples of both genera were collected. The EOs were obtained by steam distillation, and their chemical composition and bioactivity were evaluated. The results showed that the yield of EOs from male and female samples were within the usual reported ranges, varying between 0.24 and 0.58% (dry basis, d.b.). However, limonene content in three locations varied between 15 and 25%, which is between 100% and 200% higher than the values usually reported for other European countries. The antibacterial activity was determined by broth microdilution and showed that gram-positive bacteria were more susceptible to the tested EOs since, in general, lower MIC values were obtained compared to gram-negatives. The EOs from location 1 (L1F) and 2 (L2M) inhibited the growth of six out of the eight clinical strains tested. Samples from location 1 were particularly effective, exhibiting MBC against two gram-negative (*E. coli* and *P. mirabilis*) and one gram-positive bacteria (*E. faecalis*). Moreover, the majority of the EOs tested showed anti-inflammatory activity. Cytotoxic effect has been demonstrated in tumor cell lines, with the best results observed against gastric carcinoma (AGS) cells (GI_50_ between 7 to 77 µg/mL). Although generally presenting higher GI_50_, most samples also inhibited the growth of non-tumoral cells, particularly hepatocytes (PLP2 cells). Therefore, its use for their anti-proliferative activity should consider specific conditions to avoid damaging normal cells. Finally, the results and conclusions obtained led to the selection of the female shrubs from location 1 (L1F) as the plant material to be propagated in order to produce plants for a future juniper crop.

## 1. Introduction

*J. communis* L. is the conifer with the widest global distribution, being one of the species with the most extensive range in the northern hemisphere [1,2]. In Spain, wild common juniper occurs mainly in the eastern region of the country, with three subspecies coexisting: subsp. *communis*, subsp. *hemisphaerica* and subsp. *alpina* [3]. It is a typical shrubby species with different growth habits, from trailing in subsp. *alpina* to the form of a small tree up to 10 m high in subsp. *communis* [4]. Moreover, it presents the particularity of being dioecious, with females being easily distinguished from males as they are the bearers of cones (named fruits or berries).

Common juniper stands out for its multiple uses, particularly as a culinary flavoring and for aromatizing beverages [2]. This plant is also important for its ancestrally attributed virtues in folk medicine and folk veterinary medicine [5,6]. In fact, essential oils (EOs) are recognized by the European Pharmacopoeia as pharmaceutical raw materials and have been investigated by many authors in relation to their composition and bioactivity.

Although natural EOs can be obtained from plants by different methods, steam distillation is currently the predominant technique used in the industry due to its simplicity and because it does not require the use of chemical products, such as solvents, frequently used in other extraction techniques.

Plant EOs are often made up of a large number of organic molecules with the vast majority belonging to the terpene family [5]. One of the present challenges of the pharmaceutical, food and veterinary industries consists in finding bioactive molecules to be used as bactericides, due to the current problem with pathogenic bacteria whose resistance to antibiotics have developed over time [7]. Additionally, it is also important to search for antioxidant and anti-inflammatory active principles of natural origin since consumers are increasingly demanding natural products as alternatives to synthetic preservatives. Taking into account that many plant EOs and/or extracts have been used since ancient times for their antimicrobial properties in food preservation and in the cure and/or amelioration of bacterial diseases, it is necessary to deepen our knowledge in order to obtain novel bioactive compounds [8,9].

Different works reported that the yield and composition of common juniper EOs can vary within the same geographical area, as different chemotypes were reported within the same country [10]. Moreover, differences in composition have been attributed to habitat factors such as altitude, climate and soil [11,12], harvesting time [13,14] and the gender of the plant, as it is a dioecious species [15].

However, despite being a species that has been widely studied in terms of its chemical composition and the bioactivity of its EOs and extracts, no references have been found on the cultivation of the species to obtain bioproducts. The works found so far have been based on obtaining wild plant material. Possibly, unlike other plants cultivated for their EOs and active principles such as rosemary, oregano, thyme, lavender and many others, the difficulty of reproduction of common juniper, its slow growth and relatively large abundance in the natural environment, may not have made its cultivation attractive. However, it is important to cultivate the species to obtain its essential oils and extracts towards a better control of their composition and quality, which cannot be fully guaranteed in wild collection. On the other hand, nowadays, another aspect of major relevance is producing biological resources through sustainable techniques, and as far as agriculture is concerned, it is necessary to use arable land under low-input criteria and preferably through the use of organic and/or biological agriculture techniques. In this sense, it is important to meet two requirements: to use land that does not compete with food production and to use species with low requirements and that are well adapted to the territory [16]. The BeonNAT project [17] has taken these requirements into account and carried out plantations of rarely used shrub species with high hardiness so that they can be planted on marginal lands where food production is not profitable. In this sense, shrub species have been identified for their good adaptation to the changing climate, their low needs and their promising chemical composition. One of these species is the common juniper (*Juniperus communis* L). This project has identified the potential lands where common juniper can be grown in Spain and has developed a method for vegetative reproduction in nurseries, with the aim of establishing a pilot plantation of two hectares.

The objective of this study is to evaluate the yield, chemical composition and bioactivity of the essential oil from the foliage of wild common juniper collected from locations near the site where the species is going to be planted in an experimental plot. The results would help to select the most suitable origin to obtain vegetal material to produce plants for the experimental juniper crop.

## 2. Results

### 2.1. Yield of Steam Distillation and Chemical Composition of Essential Oils

The yields obtained from distilling the foliage from different locations ranged between 0.24 and 0.58% d.b. (Table 1).

When comparing the steam distillation yields of males from the different locations, significant differences were observed. Using Fisher’s Least Significant Difference (LSD) analysis, three significantly different groups were highlighted, namely, a—L1M, b—L3M and c—L2M, L4M.

With respect to the yields from female samples, and applying the same methodology, all were significantly different. Similarly, significant differences were also observed for the distillation yields of males and females from the same location, with higher values being obtained in females, with the exception of location 2.

The molecules identified according to the mass spectra and the linear retention index (LRI) and the corresponding % of the relative area are shown in Table S1 (included as Supplementary Material). A summary of the major compounds obtained for each sample, including their limit values established in the European Pharmacopoeia [18] (Ph. Eur.) and the ISO 8897:2010 [19] standard for the EO of mature berries of *Juniperus communis*, is shown in Table 1.

The results show that the compounds identified vary widely among the four locations. Particularly, the content of major compounds such as alpha-pinene, sabinene and limonene is very different: locations 1, 2 and 3 have limonene contents between 15 and 25%, exceeding the ISO and Ph. Eur. specifications, while in location 4, limonene remains within the specifications (5.94% and 7.3%), but α-pinene is below the lower limit of 20% (16.15% and 16.8%). Considering the composition, it can be observed that locations 1, 2 and 3 have a chemical profile richer in limonene, while location 4 is richer in sabinene.

### 2.2. Bioactive Properties

#### 2.2.1. Antibacterial Activity

Regarding the antibacterial potential, the results expressed as minimum inhibitory concentration (MIC) and minimum bactericidal concentration (MBC) values (%, *v*/*v*) are presented on Table 2.

The results show that none of the *J. communis* EOs were able to inhibit the growth of the gram-negative bacteria *K. pneumoniae* and *P. aeruginosa*. However, all samples presented antibacterial activity against *P. mirabilis*, with sample L4M (male from location 4) showing the strongest potential with a lower MIC value (1.25%, *v*/*v*). Both samples from location 1 (female L1F and male L1M) and L4M presented the best bactericidal activity, since the highest concentration tested of L1F was able to kill three out of the eight species assayed, while L1M and L4M were only able to kill two. On the contrary, samples from location 3 did not evidence bactericidal activity at the tested concentrations. Concerning *E. coli*, only sample L1M showed bactericidal activity; however, the bacteria was sensitive to essential oils from both genders from location 2 (L2F and L2M) and location 3 (L3M and L3F). *M. morganii* was sensitive to samples from location 1 and location 2, from both females and males. Concerning the gram-positive bacteria *E. faecalis* and methicillin resistant *S. aureus,* these were sensitive to all samples, highlighting the samples from location 1 which presented lower MIC values (1.25 %, *v*/*v*). *E. faecalis* was killed by samples from location 1 (L1F and L1M), location 2 (L2F and L2M) and by males from location 4 (L4M) (MBC = 2.5%, *v*/*v*). Regarding *L. monocytogenes*, only samples from location 1 (L1F and L1M) and males from location 2 (L2M) showed inhibiting potential. Overall, there was not a marked difference between the male and female results for each location. In general, the gram-positive bacteria presented lower MIC values, being the most susceptible strains when compared with the gram-negative bacteria (with an exception for sample L4M that had the lower MIC value for *P. mirabilis*, as already mentioned). Altogether, the most promising samples regarding antibacterial activity are the ones from location 1 and location 2, since they exhibit an inhibiting potential against three gram-negative bacteria (*E. coli*, *M. morganii* and *P. mirabilis*) and three gram-positive bacteria (*E. faecalis*, *L. monocytogenes* and MRSA). In particular, better results were obtained for samples from location 1 that exhibited MBC for two gram-negative bacteria (*E. coli* and *P. mirabilis*) and one gram-positive bacteria (*E. faecalis*).

#### 2.2.2. Antioxidant, Cytotoxicity and Anti-Inflammatory Activities

The reducing power and cellular antioxidant activity assays were used to evaluate the antioxidant activity of *J. communis* EOs. According to Table 3, all the tested samples showed antioxidant effects, with the best results being obtained for samples from location 4 (L4M and L4F) and males from location 2 (L2M) and location 3 (L3M), which present lower EC_50_ values. On the other hand, regarding the CAA assay, none of the samples exhibited a capacity to inhibit the cellular oxidation process.

Table 3 also presents the results of cytotoxicity in tumor and non-tumor cells and anti-inflammatory activity.

According to the obtained results, the gastric carcinoma (AGS) was the most susceptible cell line to *J. communis* EOs, presenting GI_50_ values ranging from 7 to 77 μg/mL. Samples from location 1 and location 4 showed the highest anti-proliferative activity.

The growth inhibition of colon (CaCo2) and lung (NCI-H460) tumoral cell lines was also higher for samples from location 1, evidenced by the results of the female in location 1 (L1F) for CaCo2 inhibition and the male (L1M) for NCI-H460 inhibition, which exhibit the lowest GI_50_ values in a range of 91 to 190 μg/mL and 59 to 287 μg/mL, respectively. Concerning breast carcinoma (MCF-7), GI_50_ values ranged from 13 to 148 μg/mL, with L1M, L2F and L3F samples showing the most promising results.

Regarding non-tumor cells, for the porcine liver primary culture (PLP2), the EOs revealed some toxicity at the active concentrations needed to inhibit the tumor cells. Nevertheless, these samples can potentially be used in some of the tumor cell lines, because the toxic concentration of PLP2 and VERO cells is higher than the one needed to inhibit the tumor cell lines.

Furthermore, the GI_50_ values required to inhibit the tumor cells AGS, CaCo2 and MCF-7 are significantly lower than the ones needed to inhibit the monkey kidney non-tumor cells.

*J. communis* EOs showed higher anti-inflammatory activity for the female from location 2 (L2F), followed by the female from location 3 (L3F) and the male from location 1 (L1M). According to Tukey’s HSD test results, only samples from location 4 did not present a significative difference between the male and female plants. The female sample from location 1 (L1F) did not evidence anti-inflammatory activity at the tested concentrations.

## 3. Discussion

### 3.1. Yield of Steam Distillation

The obtained essential oil yields are within the usual limits reported in the literature. In this work, the distillation was carried out in a pilot plant with steam distillation, whereas most values reported were obtained in the laboratory using a Clevenger system. In this sense, while in the laboratory, values higher than 1% (dry basis) are often obtained, in industrial steam distillation, the yields are usually below 1% [20]. According to a recent study conducted in Albania, soil factors such as pH value and humus content were the most significant factors impacting in the essential oil yield for juniper berries [12]. However, the pH range studied was always basic, and no acid sites were investigated. In our study there are acid and basic soils, and no correlation between soil reaction and essential oil yield has been identified.

### 3.2. Chemical Composition of Essential Oils

The chemical composition of *J. communis* EOs has been extensively studied, with many papers available, particularly on berry composition [12,13,21,22,23] but also on foliage composition [10,13,14,15]. Previous studies reported significant differences between male and female plants from the same location, which is not surprising given the wide variation in oil composition that can occur between individual plants [24]. Radoukova et al. [25] conducted a study of foliage composition collected in the summer of 2016 in three Eastern European countries where α-pinene and sabinene were the dominant constituents in the EOs of *J. communis*, being terpinen-4-ol, the third major constituent. The same conclusion was obtained by Butkienė et al. [10] for Lithuania. However, in the present work, limonene has an important role in the composition of three of the four provenances investigated, being the major constituent in females of location 2 (L2F) and 3 (L3F) and giving the oil a characteristic lemon-like aroma. In this regard, Radoukova et al. [25] reviewed the composition of *J. communis* EOs reported in different parts of the world, describing that α-pinene, sabinene and limonene are the most variable monoterpenes in several populations of *J. communis*. These variations can occur as a result of the different collection sites and subsequent dissimilar edaphoclimatic conditions, which consequently influence the essential oil composition [26].

### 3.3. Bioactive Properties

#### 3.3.1. Antibacterial Activity

According to Sela et al. [27] and Haziri et al. [28], the EOs from *J. communis* leaves and berries showed moderate to high activities against *S. aureus* (125 µg/mL) and *E. coli* strains, using the broth dilution method. Using a different method, Raina et al. [29] also reported antibacterial activity against *S. aureus* and *E. coli*, while *K. pneumoniae*, *P. aeruginosa* and *P. mirabilis* were resistant to juniper EOs. On the contrary, Angioni et al. [22] showed that the antibacterial activity of *J. communis* (berries and leaves) EOs was generally nonsignificant against *S. aureus* and *E. coli* at the highest concentration tested (MIC and MBC > 900 µg/mL).

According to Rostaefar et al. [15] who studied the chemical composition of the foliage of male and female samples from the *J. communis* subsp. Hemisphaerica, the chemical composition is affected by the environmental factors arising from seasonal variations, since some compounds may be accumulated at a particular period to respond to environmental changes. These factors can have a great influence on the differences observed for the antibacterial activity obtained for each EO [30].

#### 3.3.2. Antioxidant, Cytotoxicity and Anti-Inflammatory Activities

In the study from Xavier et al. [31], two samples of *J. communis* EOs (obtained from crown biomass) from two locations were analyzed, showing EC_50_ values in the reducing power assay between 0.97 ± 0.01 mg/mL to 1.35 ± 0.20 mg/mL, which are values similar to those observed in this work (0.98 ± 0.02 to 1.83 ± 0.03 mg/mL). Using the ORAC (oxygen radical absorbance capacity) assay, Mediavilla et al. [20] found that the EO from the twigs of *J. communis* presented similar ORAC values to those of *Eucalyptus* and *Pinus pinaster* EOs and confirm the antioxidant potential of this species, also previously described by other authors [24]. On the other hand, regarding the CAA, none of the samples exhibited a capacity to inhibit the cellular oxidation process at the maximum concentration tested. To the best of the authors’ knowledge, there are no reports in the literature describing, specifically, the cellular antioxidant activity of *J. communis* EOs.

Concerning cytotoxicity, the EOs evaluated by Xavier et al. [31] presented GI_50_ values of 44.87 ± 3.42 and 41.99 ± 3.60 μg/mL, using the NCI-H460 cell line. The results against the growth of NCI-H460 represented the best results, although the EOs proved to be cytotoxic for the remaining tumor cell lines (AGS, CaCo2 and MCF-7) at higher concentrations. In that study, EOs were hepatoxic at concentrations of 241.58 ± 9.52 μg/mL and 212.03 ± 23.26 μg/mL in PLP2 cells. Additionally, while one of the samples demonstrated to be toxic in Vero cells at a concentration of 240.73 ± 21.32 μg/mL, the other did not show toxicity (>400 μg/mL).

In general, in the present work, all samples showed cytotoxicity in the non-tumor cell lines. However, the value of GI_50_ generally was higher than that of the tumor cell lines, meaning that the EOs from location 1, 2 and 3 could potentially be used in AGS and MCF-7 cell lines and the EO from location 4 in the AGS cell line, without exhibiting relevant toxicity. Additionally, it should be noted that in vivo studies are necessary to confirm the toxicity of these oils for particular uses.

Concerning anti-inflammatory activity, with the exception of the sample L1F, all the remaining tested EOs demonstrated anti-inflammatory properties, with IC_50_ ranging from 81±1 μg/mL to 228 ± 6 μg/mL. In the previous study of Xavier et al. [31], all tested oils demonstrated anti-inflammatory properties, with IC_50_ ranging from 23.98 ± 0.92 μg/mL to 84.80 ± 1.43 μg/mL. The anti-inflammatory activity of *J. communis* berry EOs was also demonstrated by Han & Parker [32] in human dermal fibroblasts, with the anti-inflammatory activity being correlated with the major compound in the EO (α-pinene). Nevertheless, this correlation was not evidenced in the present study. Therefore, more studies on the mechanism of the action, safety and efficacy of *J. communis* EOs are recommended.

#### 3.3.3. Bioactivity Summary

In order to highlight the most promising EOs in terms of bioactive potential towards selecting plant material for crops’ establishment, each sample was qualitatively scored according to the different bioactivity assays performed, taking into account the major activities of each EO.

Therefore, qualitative scales were defined as follows: To classify the antibacterial potential, each sample was scored with “+” if the maximal MIC, from at least one strain, was 2.5% (*v*/*v*) or “++” if the MIC was equal to or lower than 1.25% (*v*/*v*). When the sample presented MBC, an additional signal “+” was added. Concerning the antioxidant activity, since no inhibition of the oxidation was observed in the CAA assay at the maximal concentration tested, only the results from the reducing power assay were taken into account. Hereupon, when the effective concentration to inhibit 50% of the oxidation was higher than 1 mg/mL, a “+” was given, and a “++” was given when the EC_50_ was equal to or lower than 1 mg/mL. To evaluate the magnitude of cytotoxicity and anti-inflammatory activity, if the concentration range was between 100 and 300 μg/mL, a “+” was given; if it was between 50–100, a “++”; and if it was under 50 μg/mL, a “+++” was given. In the case of no activity, “na” was presented. For hepatotoxicity, the same ranges used in cytotoxicity were used, but “−” signals were ascribed. Therefore, the greater the number of + signals and the lower the number of “−” signals shown in Table 4, the more potential the EO evidenced for being used as crop plant material.

## 4. Materials and Methods

### 4.1. Plant Material and Essential Oils Extraction

**Figure 1:**
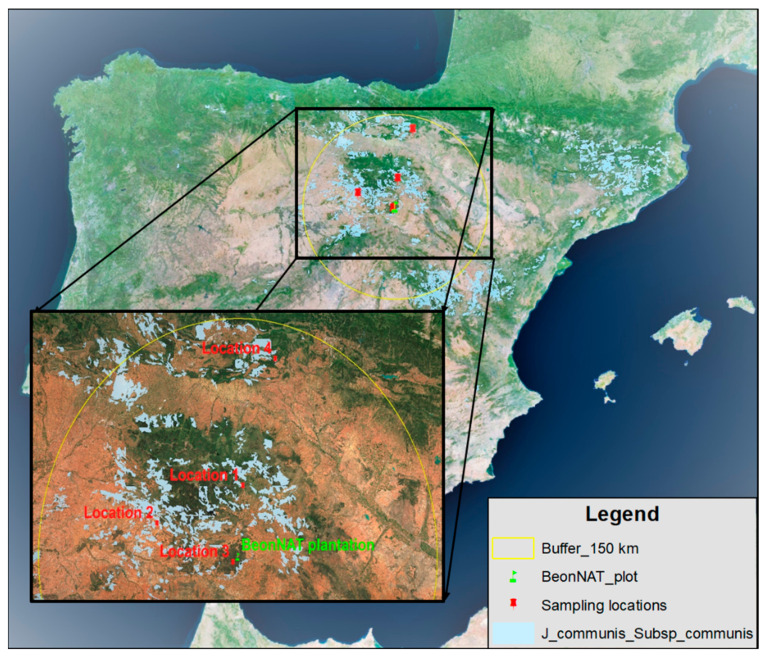
Map showing the areas where the subspecies communis can be found, the locations where plant material samples were taken from and the location of the future BeonNAT project plantation.

In order to select plant material of the species to be propagated in nurseries and subsequently undertake a plantation, a search for strands of common juniper (*Juniperus communis* L. subsp. *communis*) was carried out in the areas of origin near the point of future implantation in the town of Lubia, in the province of Soria (Spain). In Spain, the subspecies communis is located in the northern half and mainly in the center and east regions. Four sites located at a straight-line distance of no more than 150 km from the location of the future BeonNAT plantation were selected, as shown in Figure 1. Details of the collection points are given in Table 5.

A protocol for the identification and collection of the samples was written and followed in each location. A professor from the Forest Botany Unit of the Polytechnical University of Madrid participated in the identification and collection of the samples, and voucher specimens were deposited in their Unit with reference the M-BeonNAT. The samples were separately taken from males and females in several shrubs and included foliage biomass as follows: branches below 5 cm diameter, twigs, leaves and galbuli (female cones, called also berries). Samples were collected in the early autumn from 25th September 2020 until 19 October 2020.

At the laboratory, samples were cut into pieces of maximum 10 cm length and were placed in perforated boxes in a ventilated shaded place for air drying until the moisture contents were below 15% w.b. in the thickest pieces. The air-dried biomass from males and females was ground in a grinder (90 kW, slow rotating single-shaft type, 70 r.p.m, SILMISA, Onil, Spain) to a size of 30 mm. Then, it was subjected to steam distillation in a pilot distillation unit consisting of a 50 L stainless steel still connected to an electric steam boiler (ETE, Madrid, Spain). Air-dried biomass batches of 15 kg were used with three replicates per location and gender, with a duration of 2 h per extraction, a temperature inside the distiller of 98 ºC and an average steam flow rate of 10 kg/h. The distillation time was determined by considering the moment when the first drop of distillate fell. For the separation of the essential oil and the hydrolate, a glass Florentine flask was used, performing the phase separation by density difference. The oil samples were weighed, and the presence of water was eliminated by anhydrous sodium sulphate followed by filtration. The distillation yield was calculated as a percentage (*w*/*w*) on dry biomass.

To check if there are significant differences between the yields of the different locations and between males and females of the same location, different analyses of variance (ANOVA) were carried out. Significant differences were considered to exist when the *p*-value of the F-test was less than 0.05.

### 4.2. Chemical Composition of Essential Oils

The essential oils were analyzed by Gas Chromatography—Mass Spectrometry (GC-MS) on a Shimadzu GC-2010 Plus chromatograph equipped with AOC-20iPlus automatic injector (Shimadzu, Kyoto, Japan) and an SH-RXi fused-silica column (30 m × 0.25 mm i.d., film thickness 0.25 μm; Shimadzu, USA), according to the conditions described by Sprea et al. 2020 [33]. The analysis employed an oven temperature program of 40 °C for 4 min, followed by a gradual increase of 3 °C/min to 175 °C, then a rapid increase of 15 °C/min to 300 °C, held for 10 min. The injector temperature, transfer line and ion source were set at 260 °C, 280 °C and 220 °C, respectively, with an ionization energy of 70 eV. The scan range was set to 35–500 u with a scan time of 0.3 s. The essential oil was diluted in HPLC grade n-hexane (1:100) and injected with a split ratio of 1:20. Compounds were identified by comparing their mass spectra with NIST17 data and by calculating the linear retention index (LRI) based on the values obtained with a mixture of n-alkanes (C8–C40, ref. 40147-U, Supelco, Bellefonte, PA, USA) analyzed under identical conditions. Quantification was performed using the relative peak area values obtained directly from the total ion current (TIC) values, and the results were expressed as the relative percentage of total volatiles.

### 4.3. Bioactivity

In this study, the essential oils were tested for their antibacterial activity, using the broth microdilution method against clinical bacteria, their antioxidant potential, evaluated through the reducing power (RP) and through the cellular antioxidant activity (CAA), their anti-inflammatory capacity and finally their cytotoxicity in human tumor cells and in non-tumoral cells.

#### 4.3.1. Antibacterial Activity

The antibacterial activity was evaluated through the microdilution method, according to Pires et al. [34] and Falcão et al. [23], with adaptations. The bacterial strains were clinical isolates obtained from patients hospitalized at the Hospital Center of Trás-os-Montes and Alto Douro (Vila Real, Portugal), namely, five gram-negative bacteria (*Escherichia coli*, *Proteus mirabilis*, *Klebsiella pneumoniae*, *Pseudomonas aeruginosa* and *Morganella morganii*) and three gram-positive bacteria (*Enterococcus faecalis*, *Listeria monocytogenes* and methicillin-resistant *Staphylococcus aureus* (MRSA)).

The microorganisms were cultured at 37 °C in a fresh medium appropriate for maintaining exponential growth phase, for 24 h before analysis. The samples were serially diluted to obtain concentration ranges of 2.5 % (*v*/*v*) to 0.078 % (*v*/*v*). Several controls were prepared, including two negative controls (MHB with Tween 80, and another with extract) and two positive controls (MHB with Tween 80 and each inoculum and culture medium with antibiotics and bacteria). Each bacterial inoculum was pipetted into corresponding wells of a 96-well microplate, which contained 1.5 × 10^8^ Colony Forming Units (CFU)/mL. The microplates were incubated for 24 h at 37 °C on a stirring board. MIC values were determined by adding 50 μL of 0.2 mg/mL p-iodonitrotetrazolium chloride (INT) to each well, followed by incubation at 37 °C for 30 min. To determine the MBC, 10 μL from each well that showed no color change was plated onto a solid blood agar medium (7% sheep blood) and incubated at 37 °C for 24 h. The MIC was defined as the minimum concentration that inhibits visible bacterial growth, as determined by a color change from yellow to pink when microorganisms remain viable. The MBC was defined as the lowest concentration required to kill bacteria. Both MIC and MBC were expressed in % (*v*/*v*) of the EO.

#### 4.3.2. Antioxidant Activity

The antioxidant capacity was evaluated through the reducing power (RP) and the cellular antioxidant (CAA) assays. RP was performed using an ELX800 microplate reader (Bio-Tek Instruments, Inc.; Winooski, VT, USA). A volume of 0.5 mL of each concentration of the EOs (at a maximum concentration of 80 mg/mL dissolved in methanol and serial diluted) was mixed with sodium phosphate (200 mmol/L, pH 6.6, 0.5 mL) and potassium ferricyanide (1% (*w*/*v*), 0.5 mL). The mixture was incubated at 50 °C for 20 min and trichloroacetic acid (10% (*w*/*v*), 0.5 mL) was added. The mixture (0.6 mL) was transferred into the wells of a 48-well microplate in duplicate, as also deionized water (0.6 mL) and ferric chloride (0.1% *w*/*v*, 0.12 mL). The reducing power was evaluated by measuring the capacity of the extract to reduce Fe^3+^ to Fe^2+^ by measuring the absorbance at 690 nm. The results were expressed as EC_50_ values (mg/mL), corresponding to the sample concentration with 0.5 of absorbance [35].

For CAA, the determination of intracellular reactive oxygen species (ROS) was determined following the method of Wolfe and Liu [36], with modifications. To this purpose, RAW macrophage cells, obtained in ECACC, were incubated with antioxidant compounds and azobis (2-methylpropionamidine) dihydrochloride, 98% (AAPH), a compound that causes oxidative stress in order to promote the formation of free radicals. Dichloro-dihydro-fluorescein diacetate (DCFH-DA) was used as a fluorescent marker [36,37]. After RAW cells reached confluence in the culture flasks, they were washed twice with a sterile Hank’s balanced saline solution (HBSS) (pH 7.4) and then separated from the surface using 0.05% trypsin-EDTA. Cells were plated (3.0 × 10^4^) in a 100 μL of cell/well culture medium in 96-well black background culture plates and incubated until confluency (24–48 h). The perimeter wells were left empty to reduce any variation due to the location of the plate. The growth medium was removed after confluence, and the cells were then washed with HBSS. Then, 200 μL of different concentrations of the essential oils was added to each well in triplicate, with 50 μM DCFH-DA prepared in ethanol and diluted in HBSS. As a negative control, 200 μL of DCFH-DA applied in triplicate was added. Cells were incubated for 1 h at 37 °C. Quercetin was used as a positive control. Thereafter, the cells were washed 3× with HBSS to ensure that any antioxidant effects observed later in the assay were due exclusively to the compounds incorporated by the cells. Then, 100 μL of AAPH was added. Cells were immediately placed on a microplate reader (FLX800 Biotek), where real-time fluorescence was read initially and every 5 min for 40 min. Fluorescence was measured at an excitation wavelength of 485 nm and an emission wavelength of 538 nm.

Quercetin, Trypsin, 2, 7, Dichlorofluorescein diacetate (DCF-DA), Hank’s balanced saline solution (HBSS), phosphate buffered saline (PBS), and azobis (2-methylpropionamidine) dihydrochloride, 98% (AAPH) were from Sigma Chemical Co. (St. Louis, MO, USA). RAW264.7 was obtained from ECACC—European Collection of Authenticated Cell Cultures and grown in a Dulbecco’s Modified Eagle’s medium (DMEM) containing 10% heat inactivated Fetal Bovine Serum (FBS) glutamine (2 mM), penicillin (100 U mL-1) and streptomycin (100 μg/mL), at 37 °C in a humidified air incubator containing 5% CO_2_.

Regarding the quantification of *CAA*, the efficacy of the antioxidant treatments was quantified by examining the percentage reduction in fluorescence. The area under each curve was calculated using the capabilities of Excel and Integral Calculator (https://www.integral-calculator.com/, accessed on 4 November 2022). The percent reduction (or the *CAA* unit) was calculated from the formula described by Wolfe and Liu [36], shown below:

$$CAA=\%\;reduction=1-\frac{AUCsample}{AUCcontrol}\times 100$$

where *AUC* stands for “Area under the curve”.

#### 4.3.3. Cytotoxicity

For the evaluation of the cytotoxic activity of the different extracts, the Sulforhodamine B (SRB) assay was performed on different human tumor (lung carcinoma: NCI-H460, breast carcinoma: MCF-7, gastric carcinoma: AGS and colon carcinoma: CaCo2) and non-tumoral cell lines of monkey kidney (Vero) and of porcine liver primary culture (PLP2). The tumor cell lines were obtained from DSMZ—Leibniz—Institut DSMZ—Deutsche Sammlung von Mikroorganismen und Zellkulturen, while the RAW cell line was obtained from ECACC. PLP2, used for hepatotoxicity evaluation, was prepared from a freshly harvested porcine liver obtained from a local slaughterhouse. Sulforhodamine B assay (SFB, Sigma-Aldrich, St. Louis, MO, USA) was performed as previously described [38]. As a positive control, ellipticine was used [39]. For each cell line tested, the results were expressed in GI_50_ values (μg/mL), corresponding to the extract concentration that provides 50% of cell growth inhibition.

#### 4.3.4. Anti-Inflammatory Activity

The anti-inflammatory activity was determined according to the method formerly reported by Mandim et al. [40]. The nitrite concentration in the culture medium was determined to assess the capacity of the extracts to inhibit the lipopolysaccharide (LPS)-induced NO (nitric oxide) production in a murine macrophage cell line (RAW 264.7). The extracted oils, previously dissolved in DMSO/H_2_O 50:50, were re-dissolved in water at a final concentration of 8 mg/mL. The positive control used was dexamethasone, and samples without LPS were used as the negative control. The results were expressed as IC_50_ values (µg/mL), which correspond to the extract concentration responsible for 50% of NO production inhibition.

### 4.4. Statistical Analysis

All experiments were carried out in triplicate, with the results reported as average ± standard deviation. Data obtained were subjected to ANOVA (analysis of variance), and a Tukey’s HSD test was applied at *p* < 0.05.

## 5. Conclusions

Although the samples of *J. communis* EOs from different locations showed differences in the outcomes of the biological assays, those were not directly related with their different contents of major volatile compounds. Therefore, the better antibacterial activity exhibited by samples LF1 and LM4 and better antioxidant activity of samples from location 4 can possibly be attributed to differences in the whole composition, namely, regarding minor compounds that can play important roles in synergetic or additive effects. After the evaluation of the EOs’ yield, composition and bioactivity, two interesting sources were identified: females from location 1 (L1F) and males from location 4 (L4M). Although the yield is substantially higher in L4M than in L1F, the bioactivity of L4M has the drawback of its high hepatoxicity.

Therefore, location 1 was selected to obtain the plant reproduction material to be used in crop studies, especially females that will be multiplied by agamic reproduction. Location 1 has also the advantage of belonging to a geographic and climatic area similar to the location of the place available for future cultivation within the BeonNAT project.

Although common juniper essential oils generally show interesting bactericidal and cytotoxic activity, additional in vitro studies to evaluate the seasonal and environmental influence in the EOs’ composition and bioactivity are necessary. In addition, in vivo studies will also be required to confirm the toxicity of these oils for particular uses.

## Figures and Tables

**Table 1:** Essential oil yield and mean values obtained for the compounds considered in ISO 8879 and Ph. Eur. 10th edition in % of relative area.

			L1F	L1M	L2F	L2M	L3F	L3M	L4F	L4M
Compound	ISO 8897	Ph. Eur. 10th								
alpha-pinene	25–45	20–50	21.1	24.1	14.2	14.4	16.5	21.3	16.1	16.8
sabinene	4–20	0–20	10.0	4.6	1.0	0.6	4.9	2.6	20.0	20.9
beta-pinene	1–12	1–12	1.6	1.7	1.6	1.7	2.1	2.1	1.4	1.5
beta-myrcene	3–22	1–35	5.3	2.8	3.8	4.1	3.1	3.9	6.3	4.0
alpha-phellandrene	-	0–1	1.5	1.5	2.4	2.7	2.0	2.4	0.4	0.6
limonene	2–8	2–12	20.7	15.1	25.1	19.6	20.2	19.5	5.9	7.3
terpinen-4-ol	1–6	0.5–10	1.8	1.2	0.3	0.2	0.8	0.5	1.5	1.7
bornyl acetate	n.d.-0.6	0–2	0.5	0.7	0.4	0.4	0.4	0.4	0.2	0.2
beta-caryophyllene	1.5–5	0–7	5.5	3.7	3.4	1.8	2.3	2.8	4.0	1.7
alpha-humulene	1–4	-	3.8	2.5	2.2	1.0	1.5	2.0	3.0	1.1
germacrene D	1–5	-	3.8	2.8	4.0	2.7	3.5	3.5	5.7	3.5
delta-cadinene	1–3.5	-	1.7	2.4	2.3	1.7	2.8	1.7	1.9	2.6
EO YIELD										
Mean			0.351	0.298	0.428	0.571	0.301	0.24	0.579	0.533
Standard deviation			±0.010	±0.013	±0.015	±0.025	±0.020	±0.017	±0.008	±0.009

Ph. Eur. 10th—European Pharmacopoeia, 10th edition. n.d.—not detectable; EO—essential oil; L1F—female sample from location 1; L1M—male sample from location 1; L2F—female sample from location 2; L2M—male sample from location 2; L3F—female sample from location 3; L3M—male sample from location 3; L4F—female sample from location 4; L4M—male sample from location 4.

**Table 2:** Antibacterial activity of *J. communis* essential oils obtained from four different locations.

	*Juniperus communis* Essential Oils (%, *v*/*v*) ^a^																Positive Controls					
	Location 1				Location 2				Location 3				Location 4				Ampicilin		Imipenem		Vancomycin	
	Female		Male		Female		Male		Female		Male		Female		Male							
	L1F		L1M		L2F		L2M		L3F		L3M		L4F		L4M		mg mL^−1^		mg mL^−1^		mg mL^−1^	
Antibacterial Activity	MIC	MBC	MIC	MBC	MIC	MBC	MIC	MBC	MIC	MBC	MIC	MBC	MIC	MBC	MIC	MBC	MIC	MBC	MIC	MBC	MIC	MBC
gram-negative bacteria																						
*Escherichia coli*	2.5	2.5	>2.5	>2.5	2.5	>2.5	2.5	>2.5	2.5	>2.5	2.5	>2.5	>2.5	>2.5	>2.5	>2.5	<0.15	<0.15	<0.0078	<0.0078	n.t.	n.t.
*Klebsiella pneumoniae*	>2.5	>2.5	>2.5	>2.5	>2.5	>2.5	>2.5	>2.5	>2.5	>2.5	>2.5	>2.5	>2.5	>2.5	>2.5	>2.5	10	20	<0.0078	<0.0078	n.t.	n.t.
*Morganella morganii*	2.5	>2.5	2.5	>2.5	2.5	>2.5	2.5	>2.5	>2.5	>2.5	>2.5	>2.5	>2.5	>2.5	>2.5	>2.5	20	>20	<0.0078	<0.0078	n.t.	n.t.
*Proteus mirabilis*	2.5	2.5	2.5	2.5	2.5	>2.5	2.5	>2.5	2.5	>2.5	2.5	>2.5	2.5	>2.5	1.25	2.5	<0.15	<0.15	<0.0078	<0.0078	n.t.	n.t.
*Pseudomonas aeruginosa*	>2.5	>2.5	>2.5	>2.5	>2.5	>2.5	>2.5	>2.5	>2.5	>2.5	>2.5	>2.5	>2.5	>2.5	>2.5	>2.5	>20	>20	0.5	1	n.t.	n.t.
gram-positive bacteria																						
*Enterococcus faecalis*	1.25	2.5	1.25	2.5	2.5	2.5	2.5	2.5	2.5	>2.5	2.5	>2.5	2.5	>2.5	2.5	2.5	<0.15	<0.15	n.t.	n.t.	<0.0078	<0.0078
*Listeria monocytogenes*	1.25	>2.5	2.5	>2.5	>2.5	>2.5	2.5	>2.5	>2.5	>2.5	>2.5	>2.5	>2.5	>2.5	>2.5	>2.5	<0.15	<0.15	<0.0078	<0.0078	n.t.	n.t.
MRSA	2.5	>2.5	2.5	>2.5	2.5	>2.5	2.5	>2.5	2.5	>2.5	2.5	>2.5	2.5	>2.5	2.5	>2.5	<0.15	<0.15	n.t.	n.t.	0.25	0.25

^a^—EOs were tested in the concentration range of 2.5% to 0.039% (*v*/*v*). n.t.—not tested; MIC—minimum inhibitory concentration; MBC—minimum bactericidal concentration; MRSA—methicillin resistant S. aureus; L1F—female sample from location 1; L1M—male sample from location 1; L2F—female sample from location 2; L2M—male sample from location 2; L3F—female sample from location 3; L3M—male sample from location 3; L4F—female sample from location 4; L4M—male sample from location 4.

**Table 3:** Antioxidant, cytotoxic and anti-inflammatory activities of *J. communis* essential oils obtained from four different locations.

Juniperus communis									
	Location 1		Location 2		Location 3		Location 4		Control
	Female	Male	Female	Male	Female	Male	Female	Male	
	L1F	L1M	L2F	L2M	L3F	L3M	L4F	L4M	
Antioxidant activity									Trolox
Reducing power (EC_50_ mg/mL)	1.72 ± 0.06 ^b^	1.83 ± 0.03 ^a^	1.78 ± 0.02 ^a,b^	1.47 ± 0.03 ^c^	1.78 ± 0.04 ^a^	1.44 ± 0.02 ^c^	1.068 ± 0.006 ^d^	0.98 ± 0.02 ^e^	0.04 ± 0.01
									Quercetin
CAA (% oxidation inhibition) *	>2000	>2000	>2000	>2000	>2000	>2000	>2000	>2000	95.30 ± 4.60 **
Cytotoxicity GI_50_ (μg/mL)									Ellipticine (μg/mL)
AGS	7 ± 1 ^d^	18.8 ± 0.3 ^c,d^	21 ± 2 ^c^	71 ± 7 ^a,b^	61 ± 2 ^b^	77 ± 5 ^a^	9.2 ± 0.1^c,d^	11 ± 1 ^c,d^	1.23 ± 0.03
CaCo_2_	91 ± 3 ^d^	127 ± 9 ^c,d^	175 ± 2 ^a,b^	176 ± 11 ^a^	172 ± 13 ^a,b^	190 ± 5 ^a^	185 ± 19 ^a^	136 ± 7 ^b,c^	1.21 ± 0.02
MCF-7	54 ± 3 ^c,d^	13 ± 1 ^e^	20 ± 1 ^e^	70 ± 1 ^c^	22.7 ± 0.3 ^e^	127 ± 6 ^b^	47 ± 1 ^d^	148 ± 12 ^a^	1.02 ± 0.02
NCI-H460	67 ± 1 ^e^	59.0 ± 0.4 ^e^	213 ± 4 ^d^	216 ± 3 ^d^	248 ± 4 ^b,c^	228 ± 5 ^c,d^	287 ± 7 ^a^	255 ± 10 ^b^	1.01 ± 0.01
PLP2	64 ± 3 ^c,d^	58 ± 5 ^d^	71 ± 2 ^c,d^	170 ± 9 ^b^	78 ± 5 ^c^	199 ± 2 ^a^	35 ± 3 ^e^	23 ± 1 ^e^	1.4 ± 0.1
Vero	185 ± 5 ^c^	208 ± 12 ^b,c^	221 ± 3 ^a,b^	230 ± 15 ^a,b^	175 ± 2 ^c^	204 ± 10 ^b,c^	243 ± 7 ^a^	246 ± 4 ^a^	1.41 ± 0.06
Anti-inflammatory activity IC_50_ (μg/mL)									Dexamethasone (μg/mL)
RAW 264.7	>400	126 ± 8 ^c^	81 ± 1 ^d^	149 ± 1 ^b^	116 ± 3 ^c^	156 ± 2 ^b^	228 ± 6 ^a^	219 ± 4 ^a^	6.3 ± 0.4

EC_50_—effective concentration at which the absorbance is 0.5 and achieves 50% of antioxidant potential; positive control—Trolox; GI_50_—concentration of the extract causing 50% of cell growth inhibition; positive control—ellipticine; IC_50_—concentration providing 50% of inhibition of NO production in relation to the negative control (100%), dexamethasone; oils with the same letter do not present significative differences (*p* > 0.05); oils with different letters present significative differences between them (*p* < 0.05); * at the maximum concentration of 2000 μg/mL; ** quercetin—% oxidation inhibition: 0.3 μg/mL inhibits 95%; L1F—female sample from location 1; L1M—male sample from location 1; L2F—female sample from location 2; L2M—Male sample from location 2; L3F—female sample from location 3; L3M—male sample from location 3; L4F—female sample from location 4; L4M—male sample from location 4; CAA—cellular antioxidant activity; AGS—gastric carcinoma; CaCo2—colon carcinoma; MCF-7—breast carcinoma; NCI-H460—lung carcinoma; PLP2—porcine liver primary culture; Vero—non-tumoral cell lines of monkey kidney; RAW 264.7—murine macrophage cell line.

**Table 4:** Bioactivity scoring of *J. communis* essential oils obtained from four different locations. Based on a qualitative assessment.

Sample	Bioactivity				
	Antibacterial	Antioxidant	Cytotoxicity	Hepatoxicity	Anti-Inflammatory
L1F	+++	+	+++	− −	na
L1M	++	+	+++	− −	+
L2F	++	+	+++	− −	++
L2M	++	+	++	−	+
L3F	+	+	+++	− −	+
L3M	+	+	++	−	+
L4F	++	++	+++	− − −	+
L4M	+++	++	+++	− − −	+

na—no activity; L1F—female sample from location 1; L1M—male sample from location 1; L2F—female sample from location 2; L2M—male sample from location 2; L3F—female sample from location 3; L3M—male sample from location 3; L4F—female sample from location 4; L4M—male sample from location 4.

**Table 5:** Main features of sampling locations of plant material.

	Location 1	Location 2	Location 3	Location 4
Reference Male Sample	L1M	L2M	L3M	L4M
Reference Female Sample	L1F	L2F	L3F	L4F
Municipality/province	La Póveda/SO	Espeja/SO	Almazán/SO	Galbarra/NA
Longitude	41°59′48.1″ N	41°47′4.5″ N	41°33′50.9″ N	42°42′51.8″ N
Latitude	2°27′20.5″ W	3°14′4.6″ W	2°32′55.4″ W	2°9′5.5″ W
Altitude (m)	1407	1037	1080	932
Rainfall (mm)	810	592	550	807
Mean Annual T (°C)	8.0	10.0	10.0	11.2
Soil reaction	Acid	Acid	Acid	Alkaline
Sampling Date	21/09/2020	23/09/2020	14/10/2020	19/10/2020

L1F—female sample from location 1; L1M—male sample from location 1; L2F—female Sample from location 2; L2M—male sample from location 2; L3F—female sample from location 3; L3M—male Sample from location 3; L4F—female sample from location 4; L4M—male sample from location 4.

## Data Availability

The data leading to the results of this work are available from the authors upon reasonable request.

## Supplementary Materials

The following supporting information can be downloaded at: https://www.mdpi.com/article/10.3390/molecules28114448/s1, Table S1: Chemical composition of *Juniperus communis* essential oils obtained from 4 different locations. Ref. [41] is cited in the Supplementary Materials.

## Sample Availability

Samples of the essential oils are available from the authors upon reasonable request.
