# Supplementary material for: Yield, Chemical Composition and Bioactivity of Essential Oils from Common Juniper (Juniperus communis L.) from Different Spanish Origins
Source: Molecules. 2023 May 30;28(11):4448. doi: 10.3390/molecules28114448 (PMC10254527; doi:10.3390/molecules28114448)
Supplement: Supplementary file 1 [file molecules-28-04448-s001.zip › molecules-2375545-supplementary.pdf]

Table S1- Chemical composition of *Juniperus communis* essential oils obtained from 4 different locations.

**Nº**	**RT (min)**	**Compound**	**LRI [a]**	**LRI [b]**	**Relative % [c]**							
					Location 1		Location 2		Location 3		Location 4	
					Female	Male	Female	Male	Female	Male	Female	Male
					L1F	L1M	L2F	L2M	L3F	L3M	L4F	L4M
1	13.374	tricyclene	916	921	0.085±0.001	0.11±0.01	0.06±0.01	0.055±0.003	0.06±0.01	0.098±0.005	0.06±0.01	0.06±0.01
2	13.696	alpha-thujene	922	924	0.87±0.02	0.42±0.01	0.07±0.01	0.048±0.001	0.496±0.003	0.3±0.01	2.5±0.1	2.76±0.04
3	14.060	alpha-pinene	929	932	**21.09±0.43**	**24.1±0.6**	**14±1**	**14.4±0.4**	**16.5±0.9**	**21.3±1.3**	**16.1±0.1**	**16.8±0.5**
4	14.695	alfa-fenchene	941	945	0.033±0.002	0.059±0.003	0.059±0.005	0.0038±0.003	0.06±0.01	0.07±0.01	0.029±0.003	0.05±0.01
5	14.742	camphene	942	946	0.19±0.01	0.2083±0	0.179±0.004	0.16±0.02	0.21±0.02	0.35±0.001	0.15±0.02	0.15±0.01
6	15.055	thuja-2.4(10)-diene	948	953	0.06±0.01	0.126±0.001	0.043±0.004	0.0330±0.0004	0.063±0.002	0.064±0.007	0.028±0.002	0.05±0.01
7	16.083	sabinene	968	969	**10±0.18**	**4.57±0.05**	0.97±0.07	0.58±0.02	**4.9±0.1**	2.64±0.02	**20±0.2**	**20.9±0.7**
8	16.210	beta-pinene	970	974	1.631±0.002	1.703±0.002	1.6±0.1	1.71±0.01	2.11±0.07	2.1±0.04	1.4±0.01	1.5±0.01
9	17.070	beta-myrcene	987	988	**5.26±0.05**	2.83±0.01	3.8±0.2	4.13±0.03	3.051±0.002	**3.88±0.01**	**6.29±0.04**	**3.95±0.01**
10	17.497	δ-2-carene	995	1001	0.26±0.01	0.3±0.01	0.52±0.03	0.45±0.01	0.29±0.01	0.35±0.01	0.07±0.01	0.151±0.002
11	17.687	alpha-phellandrene	999	1002	1.486±0.005	1.49±0.01	2.4±0.1	2.66±0.03	1.987±0.0002	2.38±0.01	0.36±0.02	0.61±0.02
12	17.988	δ-3-carene	1004	1008	0.3±0.01	0.451±0.009	0.72±0.04	0.383±0.009	0.7979±0.0004	0.841±0.009	0.316±0.004	0.66±0.01
13	18.335	alpha-terpinene	1011	1014	0.672±0.002	0.39±0.01	0.13±0.01	0.07±0.01	0.46±0.01	0.22±0.01	0.91±0.02	1.11±0.02
14	18.762	p-cymene	1019	1020	1.44±0.01	1.498±0.002	1.5±0.1	1.58±0.03	2.191±0.001	1.96±0.03	0.69±0.03	1±0.02
15	19.087	limonene	1024	1024	**20.74±0.26**	**15.1±0.1**	**25±1**	**19.64±0.02**	**20.2±0.3**	**19.5±0.3**	5.94±0.03	**7.3±0.08**
16	19.505	cis-beta-ocimene	1033	1032	-	0.016±0.001	0.019±0.001	0.016±0.002	0.012±0.001	0.032±0.005	-	0.0051±0.0002
17	20.033	trans-beta-ocimene	1043	1044	0.16±0.01	0.09±0.02	0.10±0.01	0.17±0.01	0.041±0.004	0.118±0.003	0.047±0.002	0.11±0.01
18	20.436	isopentyl butanoate	1051	1052	0.101±0.004	0.061±0.003	0.147±0.007	0.150±0.004	0.013±0.002	0.18±0.02	0.0058±0.0004	0.0259±0.0004
19	20.545	gamma-terpinene	1053	1054	1.19±0.08	0.66±0.02	0.16±0.02	0.095±0.002	0.74±0.02	0.35±0.03	1.63±0.01	1.96±0.02
20	20.963	cis-sabinene hydrate	1061	1065	0.1109±0.0002	0.081±0.006	0.034±0.003	0.019±0.002	0.05±0.01	0.087±0.001	0.25±0.02	0.31±0.02
21	22.065	alfa-terpinolene	1082	1086	1.42±0.03	1.04±0.03	1.09±0.05	0.85±0.05	1.11±0.06	0.96±0.06	1.57±0.01	1.7±0.03
22	22.674	linalool	1095	1095	0.13±0.02	0.1±0.02	0.105±0.009	0.135±0.004	0.0629±0.0002	0.1±0.01	0.039±0.003	0.04±0.01
23	22.960	isopentyl isovalerate	1100	1103	0.05±0.01	0.1±0.02	0.043±0.004	0.016±0.001	0.03±0.01	0.07±0.01	0.02±0.004	0.038±0.005

24	23.720	cis- p-menth-2-en-1-ol	1115	1118	0.129±0.001	0.12±0.02	0.1142±0.0002	0.11±0.01	0.13±0.01	0.1±0.006	0.077±0.004	0.088±0.005
25	23.950	alpha-campholenal	1120	1122	0.19±0.01	0.3±0.01	0.132±0.009	0.098±0.001	0.18±0.02	0.252±0.003	0.081±0.003	0.119±0.003
26	24.585	trans-pinocarveol	1132	1135	0.255±0.001	0.33±0.02	0.184±0.006	0.155±0.002	0.201±0.007	0.25±0.01	0.0846±0.0002	0.108±0.004
27	24.906	cis-verbenol	1139	1137	0.12±0.01	0.23±0.01	0.08±0.01	0.076±0.001	0.12±0.002	0.52±0.02	0.051±0.004	0.084±0.004
28	25.673	trans-pinocamphone	1154	1158	0.0084±0.0002	0.041±0.004	0.012±0.002	0.009±0.002	0.0134±0.0002	0.019±0.001	-	-
29	25.782	pinocarvone	1156	1160	0.021±0.001	0.04±0.009	0.016±0.001	0.009±0.001	0.021±0.002	0.03±0.01	-	-
30	26.519	terpinen-4-ol	1171	1174	1.76±0.02	1.18±0.05	0.31±0.03	0.163±0.003	0.82±0.06	0.45±0.03	1.5±0.03	1.7±0.03
31	27.177	alpha-terpineol	1185	1186	0.181±0.001	0.2±0.04	0.39±0.02	0.57±0.05	0.26±0.05	0.31±0.02	0.054±0.004	0.075±0.003
32	27.428	myrtenol	1190	1194	0.101±0.002	0.05±0.01	0.037±0.005	0.036±0.003	0.11±0.02	0.035±0.0001	0.014±0.002	0.058±0.001
33	27.445	cis-piperitol	1190	1195	0.0185±0.0003	0.13±0.02	0.0696±0.0004	0.072±0.002	0.03±0.01	0.083±0.003	0.03±0.002	0.0076±0.0001
34	28.013	trans-piperitol	1202	1207	0.046±0.002	0.088±0.007	0.037±0.004	0.03±0.01	0.051±0.005	0.05±0.01	0.023±0.002	0.034±0.005
35	28.551	trans-carveol	1213	1215	0.09±0.01	0.0802±0.0005	0.09±0.01	0.053±0.001	0.08±0.01	0.05±0.01	0.026±0.001	0.025±0.002
36	28.977	citronellol	1222	1223	0.0257±0.0002	0.148±0.003	0.30±0.01	0.29±0.01	0.06±0.01	0.07±0.01	-	-
37	29.063	cis-carveol	1224	1226	0.028±0.002	0.07±0.01	0.0413±0.0005	0.06±0.01	0.023±0.005	0.0332±0.0004	-	-
38	30.188	piperitone	1248	1249	0.04±0.01	0.046±0.008	0.047±0.004	0.043±0.003	0.053±0.007	0.041±0.007	0.0457±0.0002	0.08±0.01
39	30.490	methyl citronellate	1254	1257	0.04±0.01	0.025±0.005	0.060±0.006	0.056±0.009	0.02±0.004	0.1±0.01	0.037±0.002	-
40	31.168	p-menth-1-en-7-al	1269	1273	0.018±0.004	0.021±0.001	0.080±0.003	0.082±0.003	0.11±0.01	0.06±0.01	-	-
41	31.694	bornyl acetate	1280	1284	0.531±0.002	0.66±0.01	0.39±0.04	0.35±0.02	0.37±0.01	0.439±0.003	0.236±0.004	0.23±0.02
42	33.482	myrtenyl acetate	1319	1324	0.12±0.01	0.076±0.003	0.147±0.001	0.16±0.01	0.115±0.008	0.081±0.001	0.028±0.003	0.03±0.01
43	34.007	trans-carvyl acetate	1331	1339	0.0442±0.0004	0.031±0.004	0.044±0.004	0.061±0.005	0.034±0.004	0.038±0.009	0.035±0.008	0.034±0.004
44	34.605	alpha-cubebene	1345	1345	0.66±0.05	4.02±0.05	1.17±0.08	3.87±0.08	3.04±0.004	0.93±0.05	1±0.01	1.32±0.06
45	35.782	alpha-copaene	1372	1374	0.35±0.01	0.81±0.02	0.77±0.05	0.352±0.003	0.68±0.02	0.49±0.02	0.278±0.005	0.34±0.01
46	36.006	geranyl acetate	1377	1379		0.012±0.003	0.05±0.01	0.06±0.01	0.015±0.003	0.0094±0.0002	0.012±0.001	0.013±0.003
47	36.100	myrtanyl acetate	1379	1385	0.04±0.01	0.019±0.005	0.08±0.01	0.07±0.01	0.042±0.004	0.037±0.002	0.025±0.004	0.026±0.001
48	36.185	beta-bourbonene	1381	1387	0.061±0.002	0.066±0.006	0.14±0.02	0.12±0.02	0.07±0.01	0.073±0.004	0.068±0.003	0.06±0.01
49	36.395	isolongifolene	1386	1389		0.16±0.03	0.07±0.01	0.12±0.03	0.103±0.009	0.029±0.005	0.066±0.009	0.15±0.01
50	36.471	beta-elemene	1387	1389	0.615±0.004	0.77±0.05	0.8±0.1	0.59±0.01	0.58±0.02	0.94±0.06	0.632±0.003	0.51±0.03
51	37.099	longifolene	1401	1407	0.036±0.002	0.079±0.002	0.169±0.002	0.240±0.008	0.082±0.005	0.202±0.003	0.026±0.001	0.027±0.003
52	37.402	α-cedrene	1409	1410	0.032±0.001	0.12±0.002	0.28±0.05	0.812±0.007	0.278±0.009	0.0932±0.0004	0.279±0.009	0.44±0.07
53	37.700	beta-caryophyllene	1416	1417	**5.46±0.13**	3.7±0.1	3.4±0.2	1.79±0.03	2.3±0.08	2.81±0.04	4.02±0.02	1.67±0.01
54	38.199	cis-thujopsene	1428	1429	1.12±0.03	2.68±0.06	**4.6±0.2**	**6.01±0.03**	**5.31±0.08**	2.42±0.07	5.54±0.04	**8.93±0.07**

55	39.126	alpha- humulene	1450	1452	3.76±0.11	2.52±0.05	2.2±0.2	1.04±0.04	1.5±0.05	2.01±0.07	2.97±0.07	1.09±0.03
56	40.059	gamma-muurolene	1472	1478	0.15±0.01	0.394±0.002	0.59±0.02	0.61±0.02	0.58±0.03	0.52±0.03	0.44±0.01	0.528±0.002
57	40.268	germacrene d	1477	1484	3.77±0.08	2.8±0.1	4.0±0.2	2.686±0.001	3.51±0.09	3.47±0.09	**5.7±0.09**	3.5±0.1
58	40.487	beta-selinene	1483	1489	0.403±0.001	0.66±0.05	0.83±0.03	0.73±0.03	0.501±0.009	1.01±0.03	0.37±0.02	0.32±0.07
59	40.787	muurola-4(14).5-diene	1490	1493	0.064±0.001	0.34±0.005	0.48±0.03	0.81±0.07	0.64±0.02	0.17±0.04	0.48±0.04	0.6±0.1
60	40.854	beta-cyclogermacrane	1492	1500	0.85±0.03	0.92±0.07	1.2457±0.0003	0.92±0.03	0.89±0.08	1.42±0.04	1.27±0.06	0.87±0.04
61	41.034	alpha-muurolene	1496	1500	0.27±0.01	1.83±0.06	1.0±0.1	0.88±0.02	1.37±0.02	0.53±0.03	0.83±0.04	1.15±0.01
62	41.212	α-chamigrene	1500	1503	-	-	0.21±0.03	0.32±0.03	0.16±0.03	-	0.3±0.02	0.6±0.1
63	41.443	beta-curcumene	1506	1514	0.15±0.01	0.14±0.01	0.415±0.002	0.32±0.01	0.04±0.01	0.05±0.01	0.11±0.02	0.142±0.001
64	41.627	gamma-cadinene	1511	1513	1.95±0.05	**5.78±0.16**	**6.0±0.2**	**9.5±0.1**	2.49±0.08	**5.05±0.11**	1.49±0.03	2.06±0.09
65	41.956	delta-cadinene	1519	1522	1.72±0.05	2.36±0.13	2.3±0.1	1.69±0.05	2.77±0.04	1.72±0.04	1.91±0.03	2.58±0.07
66	42.465	alpha.-cadinene	1532	1537	0.039±0.002	0.44±0.02	0.07±0.01	0.12±0.02	0.12±0.01	0.17±0.01	0.044±0.009	0.04±0.01
67	43.359	germacrene b	1555	1559	3.85±0.11	**4.64±0.28**	**4.4±0.3**	**4.93±0.09**	**4.19±0.08**	**4.31±0.17**	**5.981±0.009**	2.42±0.02
68	43.435	nerolidol <(e)->	1557	1561	0.05±0.01	0.17±0.04	0.4260±0.0005	0.056±0.004	0.24±0.06	0.84±0.07	0.031±0.006	0.02±0.01
69	44.058	germacren d-4-ol	1572	1574	0.12±0.003	0.17±0.01	0.15±0.02	0.114±0.005	0.58±0.03	0.34±0.07	0.15±0.02	0.24±0.03
70	44.126	spathulenol	1574	1577	0.105±0.002	0.048±0.004	0.33±0.05	0.290±0.005	0.51±0.02	0.5±0.01	0.214±0.003	0.202±0.005
71	44.357	caryophyllene oxide	1580	1582	1.1±0.04	0.88±0.03	1.22±0.07	0.316±0.004	1.24±0.02	1.41±0.03	0.576±0.008	0.24±0.01
72	45.030	widdrol	1597	1599	-	0.1±0.02	0.193±0.009	0.039±0.005	0.17±0.01	0.18±0.01	0.1±0.01	0.22±0.05
73	45.087	cedrol	1599	1600	-	0.52±0.06	0.46±0.03	2.93±0.03	0.75±0.08	0.32±0.04	0.42±0.02	0.66±0.06
74	45.366	humulene oxide ii	1606	1608	0.47±0.02	0.36±0.01	0.70±0.06	0.30±0.04	0.78±0.01	0.94±0.03	0.32±0.04	0.18±0.03
75	46.051	epicubenol	1624	1627	-	0.06±0.01	0.12±0.03	0.090±0.006	0.25±0.02	0.244±0.003	0.107±0.005	0.19±0.02
76	46.520	tau-cadinol	1637	1638	0.18±0.03	0.492±0.003	1.4±0.2	2.40±0.01	1.26±0.03	1.8±0.1	0.41±0.02	0.6±0.01
77	47.027	tau-muurolol	1650	1640	0.07±0.01	0.14±0.02	0.35±0.08	0.50±0.03	0.84±0.05	0.68±0.09	0.26±0.03	0.31±0.02
78	48.547	eudesm-7(11)-en-4-ol	1690	1700	0.015±0.001	0.03±0.003	0.06±0.01	0.086±0.005	0.081±0.005	0.083±0.009	0.024±0.003	0.03±0.01
79	54.289	levopimaradiene	2033	2039	-	-	0.08±0.01	0.0364±0.0008	0.042±0.002	0.022±0.001	-	-
80	54.613	abitatriene	2068	2058	-	0.11±0.01	0.15±0.02	0.093±0.002	0.22±0.01	0.149±0.009	0.07±0.01	0.079±0.001
81	54.880	abitadiene	2098	2088	-	-	0.22±0.03	0.1660±0.0001	0.3±0.01	0.152±0.002	0.053±0.006	0.07±0.01

[a] LRI. linear retention index determined on a DB-5 MS fused silica column relative to a series of n-alkanes (C8–C40). [b] linear retention index reported in the literature (Adams. 2017) [41]. [c] relative % is given as mean ± SD
RT- Retention time
L1F- Female Sample from Location 1; L1M- Male Sample from Location 1; L2F- Female Sample from Location 2; L2M- Male Sample from Location 2; L3F- Female Sample from Location 3; L3M- Male Sample from Location 3; L4F- Female Sample from Location 4; L4M-Male Sample from Location 4.
